# The association between waist-to-height index and prostate cancer risk: evidence from the NHANES study

**DOI:** 10.3389/fnut.2025.1610288

**Published:** 2025-06-04

**Authors:** Qinghe Gao, Zongqi Lin, Yihua Zhang, Jiantong Cai, Shaopeng Li

**Affiliations:** Department of Urology, Shishi Municipal Hospital, Quanzhou, Fujian, China

**Keywords:** WWI, abdominal obesity, visceral fat, PCA, NHANES, ROC curve

## Abstract

**Background:**

Prostate cancer (PCa) is associated with obesity, especially visceral fat. WWI may reflect the distribution of body fat more accurately. However, it is unclear whether WWI is associated with the risk of developing PCa.

**Methods:**

Seventy thousand one hundred and ninety participants took part in the National Health and Nutrition Examination Survey (NHANES). The associations between WWI and PCa risk were analyzed through multiple regression. The association between WWI and prostate cancer was analyzed in subgroups using stratified factors, with interaction tests performed to evaluate the stability of this association across subgroups. In addition, restricted cubic sample plots and threshold effects were examined to further explore the nonlinear association between WWI and prostate cancer. Finally, subject work characteristics (ROC) curves were used to evaluate the accuracy of different obesity indicators in predicting PCa.

**Results:**

Multiple regression analyses revealed that those individuals with a higher WWI index had a higher prevalence of prostate cancer, while subgroup analyses and interaction tests showed that the correlation between WWI and the prevalence of PCa differed across age groups (interaction *p* < 0.05). The ROC curves showed that the predictive power of WWI for the prevalence of PCa was superior to that of traditional indicators of obesity (both *p* < 0.05).

**Conclusion:**

There was a positive and significant association between PCa risk and WWI, especially in male participants aged <60 years. Further comprehensive studies are required to confirm our findings.

## Introduction

According to the 2020 national statistics from the American Cancer Society, prostate cancer (PCa) is the most frequent cancer in males and the second most deadly cause, behind lung cancer (1). Prostate cancer incidence is rising in developed nations as the population ages (2). The development of prostate cancer is a long-term, multi-stage process, with the environment, dietary habits, lifestyle, and genetics all influencing the development of prostate cancer (3). It is implied that the incidence of prostate cancer can be effectively reduced by the early detection and exclusion of prostate cancer risk factors as well as early diagnostic puncture.

A population-based obesity estimate indicates that by 2030, about half of all adults in the United States will be obese, contributing to the global health crisis of obesity. Numerous disorders, including diabetes, cardiovascular disease, non-alcoholic fatty liver disease, and multiple cancers, are closely linked to obesity (4). Traditional obesity assessment indices, such as body mass index (BMI) and waist circumference (WC), are unsuitable for accurately differentiating between fat and muscle mass. To more accurately represent the distribution of fat in the body, a new obesity index called the weight-adjusted waist circumference index (WWI) has been developed recently. The WWI not only efficiently assesses adverse metabolic characteristics, but also resolves the problem of correlation with BMI (5). Obesity has been reported to be associated with the risk of developing limited prostate cancer, and preventive weight control programs may contribute to reducing the recurrence in patients with clinically limited prostate cancer (6). It has not been documented before, though, that prostate cancer and WWI are related.

Thus, based on the information from the NHANES, this study aimed to investigate the relationship between the prevalence of prostate cancer and WWI.

## Methods

### Study population

NHANES is a continuous nationwide survey of the United States population that provides a wealth of data on nutrition and health using complicated, multistage, and random sampling methodologies. More information is accessible on the public website, which details the continuous design of the NHANES survey. Each participant completed an informed consent form, and all study protocols were approved by the Ethics Review Board of the National Centre for Health Statistics before data collection (5).

**Figure 1:**
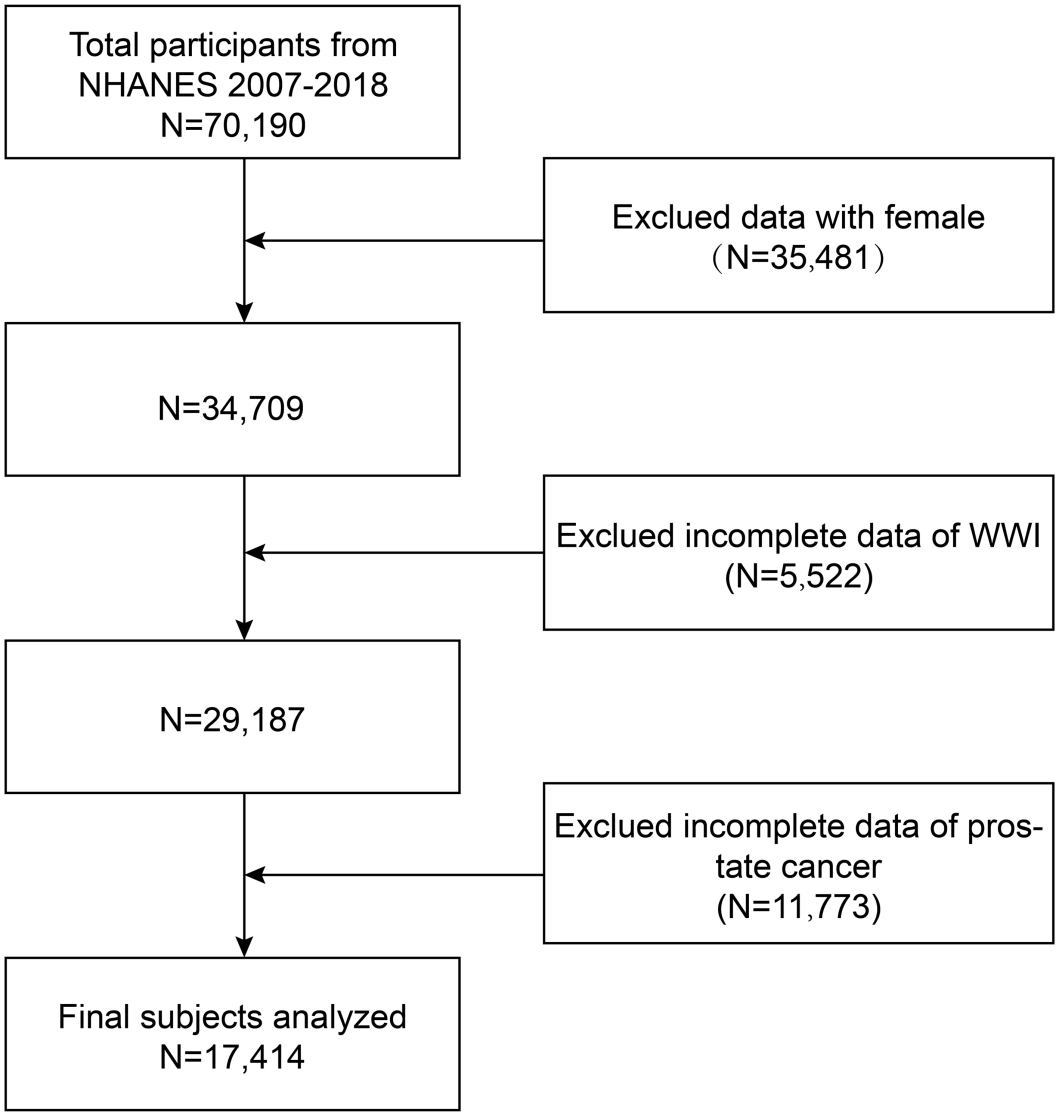
Participant selection flow chart.

In this cross-sectional study, the data from six survey cycles (two-year cycles) from 2007–2018 were used. This study excluded the data from 35,481 female participants, 5,522 participants with missing WWI data, and 11,773 participants with missing prostate cancer data from the 70,190 eligible population. A total of 17,414 participants were ultimately enrolled into the study. Figure 1 depicts the participant selection process.

### Evaluation of WWI

WWI emerges as an innovative indicator of obesity, derived by dividing WC (cm) by the square root of body weight (kg) (5). The anthropometric assessment was carried out by skilled health technicians at the Mobile Examination Centre (MEC) who closely monitored its performance through direct observation, data review, and expert examiner assessment. WWI was considered as an exposure variable in our study.

### Evaluation of PCa

In this study, previous literature was referred to and a self-administered questionnaire obtained from NHANES was used to collect information on the prevalence of prostate cancer (7, 8). The participants were required to answer the questions like “Have you ever been told by a doctor or other health professional that you have cancer or any type of malignant tumor?” and “Have you ever been told by a doctor or health professional that you have prostate cancer?” to determine prostate status (9). Prostate cancer was considered as an outcome variable in our study.

### Covariates

The potential confounders that may influence the relationship between WWI and prostate cancer were included. These variables included age, BMI, race (Mexican American, other Hispanic, non-Hispanic White, non-Hispanic Black, other races), education level (below middle school, middle school to high school, high school graduate/GED or equivalent, college or AA degree, college or AA degree or above), marital status (married, living with partner, single, divorced, widowed), poverty income ratio (PIR) (<1, ≥1), moderate recreational activity (yes, no), smoking (none, at least 100 cigarettes in life), alcohol consumption status (none, 1–3 drinks per day, 3 or more drinks per day), high blood pressure (yes, no), diabetes mellitus (yes, no or preclinical diabetes mellitus), coronary heart disease (yes, no), globulin, urinary albumin, urinary creatinine, blood albumin, blood creatinine, albumin-alanine transaminase (ALT), albumin-grass aminotransferase (AST), direct high-density lipoprotein cholesterol (HDL-C), serum cholesterol, blood glucose, glycated hemoglobin, serum iron, blood triglyceride, and blood uric acid (7, 8).

### Statistical analysis

With chi-square tests performed for categorical variables and *t*-tests performed for continuous variables, the study assessed the characteristics of the participants by grouping them into quartiles according to the WWI. Three distinct models were examined through multifactorial logistic regression to explore the relationship between prostate cancer and WWI. Model 1 did not involve any adjustment for covariates. Model 2 involved adjustments for age and race. Model 3 was adjusted for age, BMI, race, education level, marital status, PIR, moderate recreational activity, smoking status, drinking status, hypertension, diabetes mellitus, coronary artery disease, globulin, urinary albumin, urinary creatinine, blood albumin, blood creatinine, ALT, AST, HDL-C, serum cholesterol, blood glucose, glycated hemoglobin, serum iron, blood triglyceride, and blood uric acid. A trend test was conducted to examine the trend of the non-linear connection between WWI and prostate cancer after the continuous variable was converted to a categorical variable (quartiles). The association between WWI and prostate cancer was analyzed in subgroups using a series of stratified factors such as age, smoking status, BMI, diabetes mellitus, hypertension, and coronary heart disease, while interaction tests were performed to assess the stability of this association across subgroups. In addition, restricted cubic sample plots and threshold effects were examined to further explore the nonlinear relationship between WWI and prostate cancer. Finally, the accuracy of the variables WWI, BMI, WC, and height in predicting prostate cancer was evaluated against the area under the curve (AUC). R software (version 4.2.3), MedCalc software (version 19.7.4), and Empower software (version 2.0) were applied in this study to conduct all statistical analyses.

## Results

### Baseline characteristics

The study included a total of 17,414 adult American men, of whom the age range (Mean + SD) was 49.52 ± 17.74 years. In the population under study, 2.92% of the participants had prostate cancer. Table 1 illustrates the characteristics of participants stratified by WWI quartiles. The four WWI quartile ranges were Q1 (8.11–10.3), Q2 (10.3–10.8) Q3 (10.89–11.44), and Q4 (11.44–15.70). The risk of prostate cancer increased with the rising quartiles of WWI (Q1: 0.60%, Q2: 2.23%, Q3: 3.10% and Q4: 5.74%). Table 1 shows the detailed demographic data of all respondents. The results indicate that those with high WWI are more likely to be older non-Hispanic people, have hypertension, diabetes, coronary heart disease, no moderate recreational activities, alcoholics, and smokers. They tended to be accompanied by higher BMI, serum globulin, blood creatinine, blood uric acid, triglycerides, blood glucose, glycosylated hemoglobin, and urinary albumin, along with lower urinary creatinine, serum albumin, serum iron, and HDL-C (all *p* < 0.001).

**Table 1:** Table of participants’ baseline characteristics.

WWI	Q1 (8.11–10.3)	Q2 (10.3–10.8)	Q3 (10.89–11.44)	Q4 (11.44–15.70)	*p*-value
	*N* = 4,354	*N* = 4,353	*N* = 4,353	*N* = 4,354	
Age, years	36.11 ± 13.65	46.34 ± 15.30	53.39 ± 15.79	62.24 ± 14.91	<0.001
BMI (kg/m^2^)	24.67 ± 3.92	27.71 ± 4.53	29.67 ± 5.25	32.50 ± 6.82	<0.001
Globulin (g/dL)	2.80 ± 0.43	2.86 ± 0.43	2.90 ± 0.45	2.94 ± 0.46	<0.001
Albumin, urine (μg/mL)	25.78 ± 263.67	36.18 ± 284.82	48.98 ± 270.68	91.62 ± 418.48	<0.001
Creatinine, urine (mg/dL)	155.46 ± 92.90	145.53 ± 81.87	137.88 ± 78.67	132.20 ± 73.64	<0.001
Albumin, serum (g/dL)	4.44 ± 0.31	4.36 ± 0.30	4.28 ± 0.30	4.18 ± 0.31	<0.001
Creatinine, serum (mg/dL)	1.01 ± 0.32	1.01 ± 0.50	1.01 ± 0.48	1.07 ± 0.53	<0.001
ALT (U/L)	25.56 ± 14.89	30.33 ± 21.00	31.11 ± 23.46	28.42 ± 29.59	<0.001
AST (U/L)	26.77 ± 17.84	27.41 ± 14.16	27.82 ± 19.87	26.90 ± 16.99	<0.001
HDL-C (mg/dL)	52.67 ± 15.00	48.01 ± 14.08	46.55 ± 13.50	45.17 ± 12.51	<0.001
Cholesterol (mg/dL)	183.56 ± 38.01	196.98 ± 39.44	195.34 ± 43.05	185.64 ± 43.21	<0.001
Glucose, serum (mg/dL)	92.15 ± 24.37	100.99 ± 36.03	108.08 ± 42.24	116.94 ± 48.09	<0.001
Glycohemoglobin (%)	5.39 ± 0.70	5.65 ± 0.99	5.89 ± 1.17	6.19 ± 1.26	<0.001
Iron, serum (μg/dL)	17.19 ± 6.50	16.95 ± 6.02	16.40 ± 6.04	15.75 ± 5.82	<0.001
Triglycerides (mg/dL)	125.44 ± 114.08	174.93 ± 149.53	186.46 ± 158.98	187.65 ± 148.82	<0.001
Uric acid (mg/dL)	5.76 ± 1.15	6.02 ± 1.22	6.15 ± 1.31	6.28 ± 1.40	<0.001
Race, %					<0.001
Mexican American	349 (8.02%)	725 (16.66%)	830 (19.07%)	807 (18.53%)	
Other Hispanic	275 (6.32%)	385 (8.84%)	448 (10.29%)	447 (10.27%)	
Non-Hispanic White	1,652 (37.94%)	1,763 (40.50%)	1,789 (41.10%)	2,219 (50.96%)	
Non-Hispanic Black	1,489 (34.20%)	898 (20.63%)	778 (17.87%)	569 (13.07%)	
Other races	589 (13.53%)	582 (13.37%)	508 (11.67%)	312 (7.17%)	
Education level, %					<0.001
Less than junior high school	175 (4.02%)	373 (8.57%)	578 (13.28%)	803 (18.44%)	
Middle to high school	614 (14.10%)	629 (14.45%)	673 (15.46%)	660 (15.16%)	
High school graduate/GED or equivalent	1,027 (23.59%)	1,027 (23.59%)	1,070 (24.58%)	1,055 (24.23%)	
Some college or AA degree	1,351 (31.03%)	1,189 (27.31%)	1,072 (24.63%)	1,099 (25.24%)	
College graduate or above	1,187 (27.26%)	1,135 (26.07%)	960 (22.05%)	737 (16.93%)	
Marital status, %					<0.001
Married	1,698 (39.00%)	2,605 (59.84%)	2,768 (63.59%)	2,765 (63.50%)	
Widowed	46 (1.06%)	105 (2.41%)	184 (4.23%)	352 (8.08%)	
Divorced	289 (6.64%)	398 (9.14%)	401 (9.21%)	491 (11.28%)	
Separated	124 (2.85%)	108 (2.48%)	132 (3.03%)	123 (2.82%)	
Never married	1,616 (37.12%)	735 (16.88%)	532 (12.22%)	411 (9.44%)	
Living with partner	581 (13.34%)	402 (9.24%)	336 (7.72%)	212 (4.87%)	
Income to poverty ratio					0.019
<1	835 (19.18%)	728 (16.72%)	751 (17.25%)	771 (17.71%)	
≥1	3,519 (80.82%)	3,625 (83.28%)	3,602 (82.75%)	3,583 (82.29%)	
Diabetes, %					<0.001
No	4,181 (96.03%)	3,946 (90.65%)	3,592 (82.52%)	2,991 (68.70%)	
Yes	127 (2.92%)	344 (7.90%)	630 (14.47%)	1,201 (27.58%)	
Borderline	46 (1.06%)	63 (1.45%)	131 (3.01%)	162 (3.72%)	
Coronary heart disease, %					<0.001
No	4,316 (99.13%)	4,222 (96.99%)	4,086 (93.87%)	3,826 (87.87%)	
Yes	38 (0.87%)	131 (3.01%)	267 (6.13%)	528 (12.13%)	
Hypertension, %					<0.001
No	3,696 (84.89%)	3,084 (70.85%)	2,582 (59.32%)	1,958 (44.97%)	
Yes	658 (15.11%)	1,269 (29.15%)	1,771 (40.68%)	2,396 (55.03%)	
Moderate activities (%)					<0.001
No	2,479 (56.94%)	2,699 (62.00%)	2,876 (66.07%)	3,105 (71.31%)	
Yes	1,875 (43.06%)	1,654 (38.00%)	1,477 (33.93%)	1,249 (28.69%)	
Alcohol consumption, %					<0.001
No	1,056 (24.25%)	1,207 (27.73%)	1,420 (32.62%)	1,785 (41.00%)	
1–3 cups per day	2,160 (49.61%)	2,123 (48.77%)	2,031 (46.66%)	1,887 (43.34%)	
More than 3 cups per day	1,138 (26.14%)	1,023 (23.50%)	902 (20.72%)	682 (15.66%)	
Smoking status, %					<0.001
No	2,286 (52.50%)	2,119 (48.68%)	1,893 (43.49%)	1,656 (38.03%)	
At least 100 cigarettes in life	2,068 (47.50%)	2,234 (51.32%)	2,460 (56.51%)	2,698 (61.97%)	
Prostate cancer, %					<0.001
No	4,328 (99.40%)	4,256 (97.77%)	4,218 (96.90%)	4,104 (94.26%)	
Yes	26 (0.60%)	97 (2.23%)	135 (3.10%)	250 (5.74%)	

Unless specified differently, categorical variables are given as % and continuous variables as mean ± SD.

### Connection between WWI and PCa

The results of multifactorial logistic regression analysis conducted using the three models are shown in Table 2. There is a significant positive connection between WWI and PCa. The higher the WII, the higher the risk of prostate cancer. This connection was significant in our Model 1 (OR = 2.34; 95% CI = 2.08–2.6). Additionally, even after all variables were accounted for, these increases in Model 3 remained statistically significant (OR = 1.22; 95% CI = 1.0–1.46). For additional analysis, WWI was simultaneously transformed from a continuous variable to a categorical variable (quartiles). Once all confounders were taken into account, it was found out that the Q2, Q3, and Q4 groups of the WWI had an increased risk of prostate cancer compared to the Q1 group by 0.71, 0.5, and 0.87 times, respectively (Q2: OR = 1.71; 95% CI = 1.07–2.71, Q3: OR = 1.50; 95% CI = 0.94–2.39, Q4: OR = 1.87; 95% CI = 1.15–3.04).

**Table 2:** Connection between WWI and PCa in the multiple regression model.

WWI	OR (95% CI) *p*-value		
	Model 1	Model 2	Model 3
Continuous	2.34 (2.08, 2.62) < 0.0001	1.12 (0.97, 1.30) 0.1301	1.22 (1.01, 1.46) 0.0363
Categories			
Q1	Reference	Reference	Reference
Q2	3.79 (2.46, 5.86) < 0.0001	1.94 (1.23, 3.05) 0.0042	1.71 (1.07, 2.71) 0.0239
Q3	5.33 (3.49, 8.12) < 0.0001	1.64 (1.05, 2.57) 0.0291	1.50 (0.94, 2.39) 0.0913
Q4	10.14 (6.76, 15.22) < 0.0001	1.88 (1.21, 2.92) 0.0051	1.87 (1.15, 3.04) 0.0118
*p* for trend	<0.0001	0.0538	0.0350

Model 1: No covariates were adjusted.

Model 2: Adjusted for age and race.

Model 3: Adjusted for all covariates.

### Non-linear association between WWI and PCa

**Figure 2:**
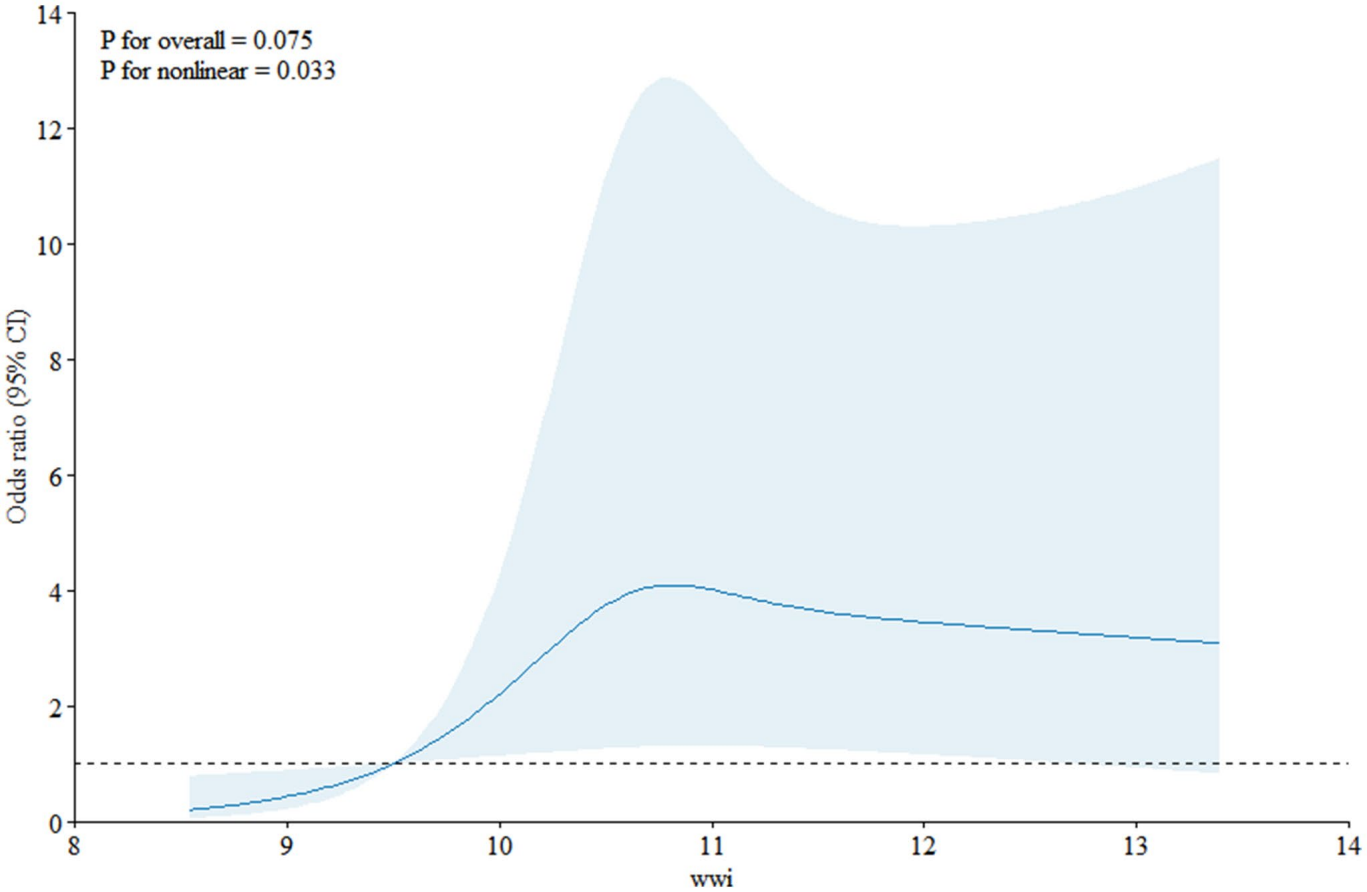
Non-linear association between WWI and PCa risk.

Restricted cubic spline (Figure 2) depicts the dose–response analysis between WWI and prostate cancer. A nonlinear positive correlation was discovered between prostate cancer risk and the increase in WWI (*p* for nonlinear = 0.033). In particular, the risk of prostate cancer rose sharply as WWI dragged on. Additionally, a segmented regression model was used to investigate the threshold effect of the curve. A nonlinear association was discovered between WWI and prostate cancer (logarithmic likelihood ratio test = 0.006), while an inflection point of the curve was noticed at 10.8 (Table 3). When WWI was less than 10.8, WWI was positively associated with prostate cancer risk (OR = 2.51; 95% CI = 1.39–4.51). However, no statistically significant positive correlation was observed when WWI was greater than 10.8 (OR = 1.04; 95% CI = 0.84, 1.30).

**Table 3:** Threshold effects of WWI and prostate cancer.

WWI	OR	95% CI	*p*-value
Inflection point (< 10.8)	2.51	1.39, 4.51	0.0022
Inflection point (>10.8)	1.04	0.84, 1.30	0.7241
Logarithmic likelihood ratio test			0.006

Adjusted for all covariates.

### Stratified analyses based on additional variables

**Figure 3:**
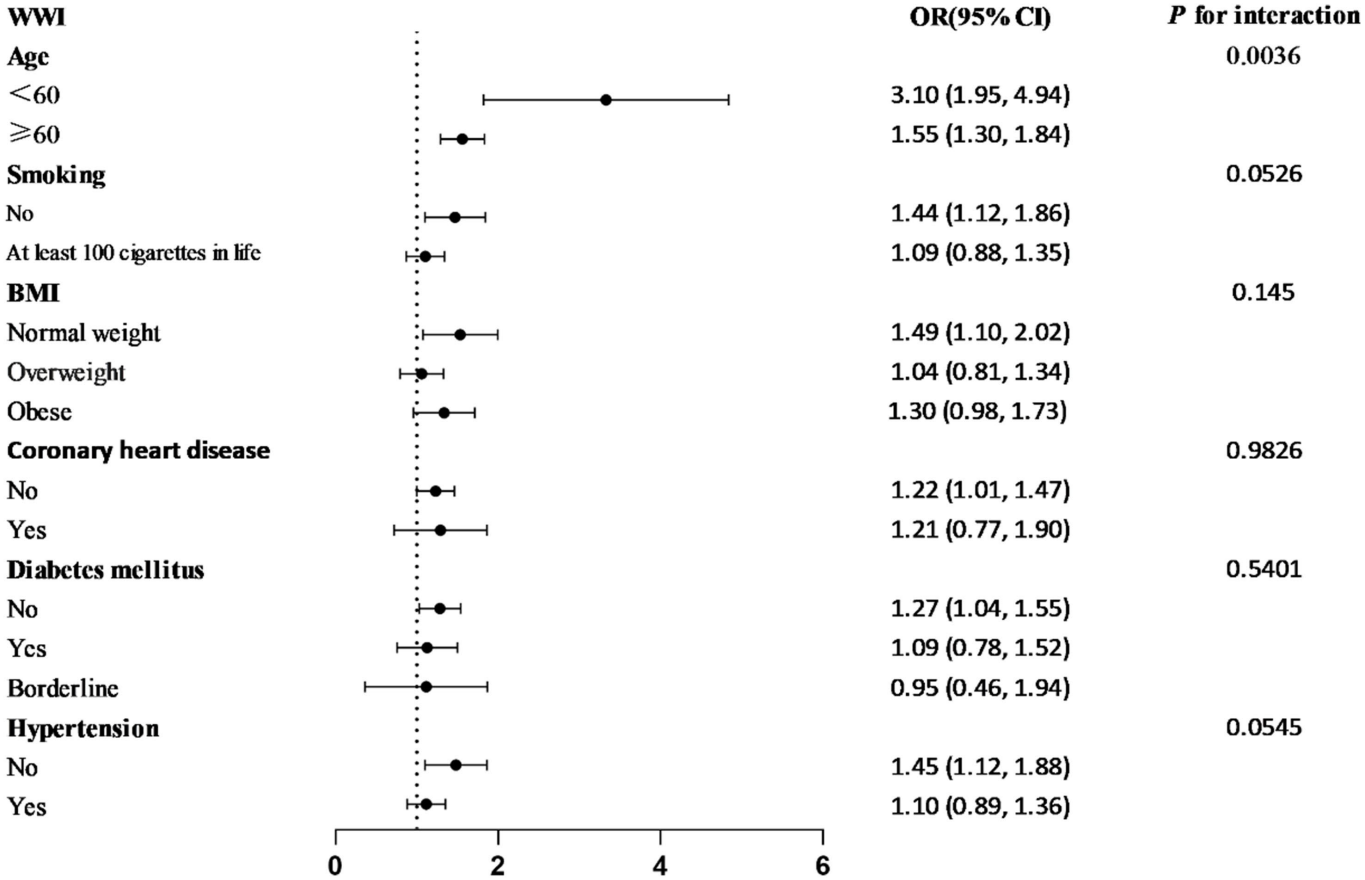
The relationship between WWI and PCa according to basic features.

The subgroup analyses stratified by smoking, age, BMI, diabetes, coronary heart disease, and hypertension were conducted to ascertain whether the relationship between WWI and prostate cancer remained constant throughout stratification (Figure 3). It was found out that the association between WWI and prostate cancer was inconsistent. Among the subgroups, the correlation between WWI and prostate cancer was higher in those who were less than 60 years old, non-smokers, and had normal weight, no diabetes, no hypertension, and no coronary heart disease. Interaction tests revealed that the relationship between WWI and prostate was independent in the smoking, BMI, diabetes, hypertension, and coronary heart disease subgroups (*p* for interaction >0.05) but was affected by the age subgroups (*p* for interaction <0.05). The young and middle-aged people with higher WWI were more likely to develop prostate cancer than older patients.

### The predictive power of different obesity indicators

**Figure 4:**
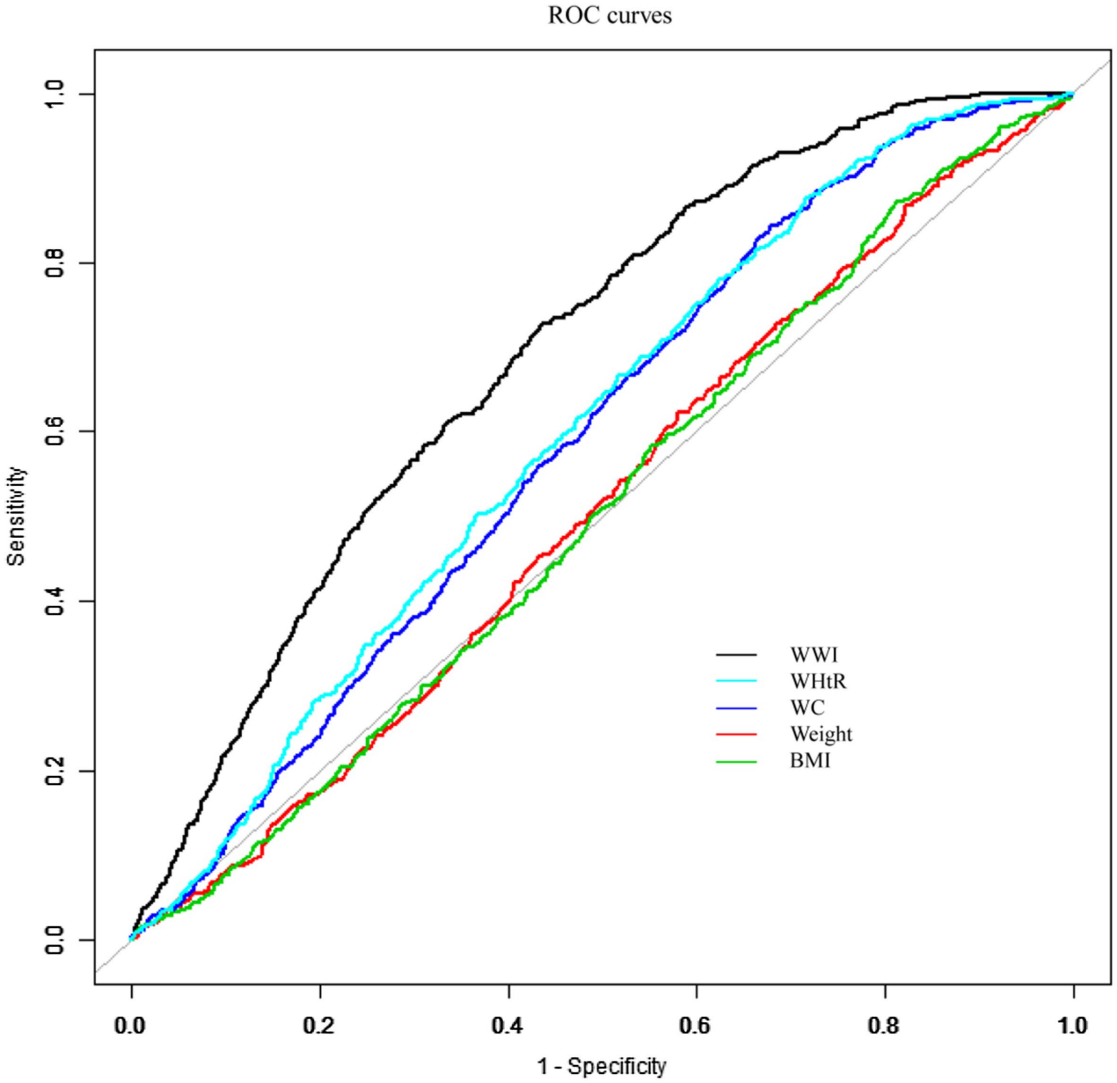
ROC curves for different obesity indicators to predict prostate cancer risk.

The AUC value was determined by fitting a ROC curve to evaluate the prediction accuracy of WWI with other conventional obesity indicators (BMI, WHTR, WC, weight) for prostate cancer (Figure 4). It was found out that WWI was much more diagnostic than other traditional indicators in predicting prostate cancer. With an ideal cut-off value of 11.0135, a specificity of 0.5666 and a sensitivity of 0.7244, the AUC was 0.693 (95% CI: 0.687–0.700) (Table 4). Additionally, the AUC values for WWI and the other obesity indices differed to a statistically significant extent (DeLong test *p*-value <0.0001).

**Table 4:** ROC curves for different obesity indicators to predict prostate cancer risk.

Obesity indicators	AUC	95% CI	Best thresholds	Specificity	Sensitivity	*Z* statistic	DeLong test
WWI	0.693	0.687–0.700	11.0135	0.5666	0.7244	Reference	Reference
WHtR	0.599	0.592–0.606	0.5283	0.2838	0.876	12.906	*p* < 0.0001
WC	0.59	0.583–0.597	93.15	0.321	0.8425	11.122	*p* < 0.0001
Weight	0.509	0.501–0.516	103.65	0.1777	0.8661	10.549	*p* < 0.0001
BMI	0.506	0.499–0.514	23.695	0.1889	0.872	15.559	*p* < 0.0001

## Discussion

In this study, the data from six cycles of the NHANES from 2007 to 2018 were used to establish the unadjusted Model 1 (OR = 2.34; 95% CI = 2.08–2.62), and Model 3 (OR = 1.22; 95% CI = 1.01–1.46) adjusted for all covariates by means of multivariate logistic regression, revealing for the first time a strong positive association between WWI and the development of prostate cancer among US adult men. To further validate the probabilistic correlation between WWI and developing prostate cancer, classified WWI was quadrupled and a trend test was conducted. The results showed that the trend *p*-value of all three models was less than 0.05, indicating the high stability of the research model. In addition, the participants were divided into two subgroups by age: age ≥60 years and age <60 years, with an interaction test performed to indicate a *p*-value of 0.0036. For every 1-unit increase in WWI among those aged <60 years, the risk of prostate cancer increased by 2.10 times (OR = 3.10; 95% CI = 1.95–4.94), which was much higher than that among those aged ≥60 years (OR = 1.55; 95% CI = 1.30–1.84). Many existing studies have demonstrated a strong correlation between age changes and prostate cancer, which may be related to the changes in androgen levels, especially testosterone levels, among male humans as they age (10–12). There is a significant correlation between the changes in testosterone concentration and the development and progression of prostate cancer (13). The endocrine system of young and middle-aged men may be in a more active state compared to older men with stable testosterone levels, possibly making them more susceptible to various hormones such as androgens, which can increase the risk of prostate cancer (14). A clinical study conducted in the United States showed that for every 10 ng/dL decrease in testosterone per year, the risk of PCa increased by 14% (OR = 1.14; 95% CI = 1.03–1.25), suggesting that a rapid age-associated decrease in testosterone was associated with an increased risk of PCa (15). This is consistent with our findings. In addition, young and middle-aged men face great social pressure and may be more susceptible to poor lifestyle habits such as diet, sleep, and smoking (16–18), which may also increase the risk of prostate cancer in young and middle-aged men.

As the study on the mechanism of obesity-induced various diseases has been increasingly concerned by various research institutes, the relationship between obesity and various cancers has also become a hot topic in current research (7, 9, 19). A cross-sectional study conducted by Liu et al. (7) suggested a strong correlation between obesity and prostate cancer. So far, many scholars have attempted to explore the mechanism of the association between obesity and prostate cancer, expecting its application to clinical treatment (6, 20). Zhao et al. (21) conducted a relevant study in an obese animal model, discovering that the chemokine CCL5, which is secreted by adipose tissues under the stimulation of chronic inflammation, may be involved in the metastasis of tumors in mice. This finding highlights the important role played by the endocrine function of adipose tissue in the association between obesity and cancer. The adipose tissue in humans can be primarily classified as either white or brown, with the white kind having endocrine functions (22). Multiple adipokines secreted by white adipose tissue in response to inflammatory stimuli are involved in inflammation and immune regulation in the body, including the high circulating levels of leptin, tumor necrosis factor-α (TNF-α), interleukin 6 (IL-6) and vascular endothelial growth factor (VEGF). These adipokines are strongly associated with increased risk and increased aggressiveness of prostate cancer, whereas the circulating levels of lipofuscin are usually low and negatively associated with disease grading of prostate cancer. In addition, adipokines can modulate prostate cancer cells and influence their biological processes such as differentiation, apoptosis, proliferation, and angiogenesis (23). Obesity induces a systemic inflammatory response through multiple mechanisms, thus enhancing the endocrine function of white adipose tissue, which is one of the important mechanisms of the association between obesity and prostate cancer. Matheson et al. (24) verified through animal experiments that the adipokines secreted by adipocytes of obese rats, such as TNF-α, IL-6, leptin, etc., are capable of triggering a systemic inflammatory response, which can provide the basis for the development and progression of prostate cancer by creating an inflammation-promoting environment. Sufficient evidence suggests that the long-term inflammatory responses throughout the body and around the prostate can lead to the development of prostate cancer (25, 26). Furthermore, a study conducted by Feng and colleagues revealed that the growth of adipose tissue surrounding the prostate, particularly in the retropubic area, maybe a significant factor in the onset and spread of prostate cancer. Their study demonstrated that the interaction between adipose tissue and the prostate through paracrine secretion of exosomes may lead to the formation and progression of prostate cancer (27).

The above studies have shown that obesity contributes to the development of prostate cancer and is related to the enhanced endocrine function of white adipose tissues under the stimulation of inflammation after obesity. However, due to the different distribution of adipose tissue, there is a diversity of indicators for describing obesity, and the traditional indicators include body mass index (BMI), waist circumference (WC), body weight, and waist-to-hip ratio (WHTR), etc. (9). The BMI not only ignores the accumulation of fat in the abdominal area, which is compensated for by the WC and WHTR, but also ignores the distribution of fat in other parts of the body. Several studies have confirmed the correlation between BMI and prostate cancer (28–30), and there has been increasing concern that increased abdominal fat deposition is a risk factor for prostate cancer (31, 32). However, the strength of the correlation with prostate cancer has not been confirmed. Currently, prostate cancer has not been differentiated in terms of the strength of the association (33). Body weight simply reflects the weight of the body rather than the fat distribution and the correlation with prostate cancer has been less well studied, with no clear correlation discovered. The newest obesity index, WWI, combines the dual advantages of body fat distribution and abdominal fat accumulation, and the comparison of the ROC curve and the area under the curve (AUC) in this study highlights the satisfactory predictive efficacy of WWI with prostate cancer (DeLong test <0.0001), which is also coherent with the inference of the results of the above studies. Prostate cancer and WWI exhibited a non-linear connection in this analysis, with an inflection point noticed at a WWI of 10.8 (Figure 2). According to the results of the threshold effect test, while every unit increase in WWI was linked to a 4% increase in the risk of prostate cancer on the right side of the inflection point, it was associated with a 151% increase on the left side of the inflection point. This finding shows that the prevalence of prostate cancer stabilizes when the WWI reaches 10.8 and that this saturation effect is suggestive of biological homeostasis in the organism (34).

In summary, this study adopted WWI as a new independent risk factor for prostate cancer in adult men in the United States, demonstrating that the new indicator of WWI combines the respective advantages of BMI and WC. To further reduce the incidence of prostate cancer in the population, it is important to focus on the degree of abdominal fat accumulation while reducing body weight to prevent and control the disease in a multidimensional manner. In addition, it provides a convenient, simple, fast, and more feasible means to predict the risk of disease through anthropometric indicators.

This is the first study to examine the connection between PCa prevalence in the US population and WWI. However, there are some limitations facing this study. Since the NHANES database does not provide pathologic or imaging confirmatory information, the respondents’ self-reported physician diagnoses are required to identify the individuals with prostate cancer. There is a potential risk of bias with this method of acquisition. If such errors are non-differential, the degree of association observed in the study can be underestimated. Secondly, despite a possible association discovered between WWI and prostate cancer, it was difficult to draw causal inferences because this study was based on cross-sectional data. Future studies may consider using the methods with more causal inference capabilities, such as prospective cohort studies, interventional trials, or relying on Mendelian randomization (MR) analyses to infer the potential causal associations between WWI and prostate cancer. In addition, the NHANES database lacks follow-up data on PCa patients. In this study, only the correlation between WWI and PCa prevalence was determined, while the progression and prognosis of PCa patients were not assessed. Finally, the confounding factors that might influence the association between WWI and prostate cancer were controlled as much as possible to improve the accuracy of the analytical results. However, it is noteworthy that some of the variables may be in the causal path and over-adjustment may lead to underestimation of the actual association.

## Conclusion

Therefore, it was concluded in this study that there is a positive and nonlinear association between WWI and prostate cancer prevalence. This association is statistically significant when WWI is less than 10.8. This trend is particularly pronounced in men aged under 60. WWI is more accurate in predicting PCa prevalence than the traditional indicators of obesity. However, further research remains necessary to validate the mechanism of association between WWI and PCa.

## Data Availability

The original contributions presented in the study are included in the article/supplementary material, further inquiries can be directed to the corresponding authors.
